# Poorer prognosis in patients with advanced gastric squamous cell carcinoma compared with adenocarcinoma of the stomach: Case report

**DOI:** 10.1097/MD.0000000000009224

**Published:** 2017-12-15

**Authors:** Ying Meng, Jiazhao Zhang, Huijun Wang, Yiping Zhang, Ruirui Sun, Zhen Zhang, Fang Gao, Chengsuo Huang, Shu Zhang

**Affiliations:** a School of Medicine and Life Sciences, University of Jinan-Shandong Academy of Medical Sciences; b Department of Medical Oncology, Shandong Cancer Hospital Affiliated to Shandong University; c Shandong University of Traditional Chinese Medicine, Jinan, Shandong Province; d Department of Medicine, Weihai Guoan Hospital; e Department of Medicine, Weihai Children's Hospital, Weihai; f Department of Oncology, Yicheng Hospital, Zaozhuang, Shandong Province, PR China.

**Keywords:** gastric cancer, overall survival, prognosis, squamous cell carcinoma

## Abstract

**Rationale:**

Primary squamous cell carcinoma (SCC) of the stomach is a rare disease. The pathogenesis and prognosis of advanced SCC remains to be elucidated. The aim of the current study was to investigate the prognosis of recurrent or metastatic SCC of the stomach.

**Patient concerns:**

A retrospective study examined the clinical characteristics and survival outcomes of 14 patients diagnosed with recurrent or metastatic SCC of the stomach, including 7 patients followed up in the hospital and 7 patients selected from the PubMed and Chinese National Knowledge Infrastructure (CNKI) database with meta-analysis between January 2003 and January 2016.

**Diagnoses:**

All patients meet the following diagnoses criteria: histological diagnosis of gastric squamous cell carcinoma; the tumor must not be located in the cardia area or extend into the esophagus; presence of local relapse or distant metastases of gastric SCC in computed tomography (CT) images; and no evidence of secondary SCC in the body. Clinical pathological data and follow-up data were obtained from the medical record or case report of each patient.

**Interventions:**

Palliative chemotherapy was administered in 14 patients diagnosed with recurrent or metastatic gastric SCC.

**Outcomes:**

The median age of 14 patients (10 males and 4 females) was 61 years old (range, 28–76). In total, 57% (8/14 cases) of tumors were located on the lesser curvature side of the stomach and 64% (9/14 cases) of metastatic sites were identified in the liver. All patients received systemic chemotherapy, and their median survival was 7.0 months (range, 2.0–22.3 months).

**Lessons:**

The median survival of patients with advanced gastric SCC was shorter than the median survival (11 months) of advanced gastric adenocarcinoma, suggesting that advanced gastric SCC may have a poorer prognosis compared with adenocarcinoma of the stomach in recurrent or metastatic stage.

## 1 Introduction

Gastric cancer (GC) is the fourth most common cancer and the fourth leading cause of cancer mortality worldwide, with the third highest incidence and mortality in China.^[1]^ In China, 400,000 new GC cases are estimated to occur annually, which constitutes 42% of all patients with GC worldwide, with 281,000 cases of mortality in China.^[1,2]^ At the time of diagnosis, 50% to 70% of these cases were already at an advanced stage.^[1–4]^ The majority of patients with GC were diagnosed as having adenocarcinoma. The clinical pathological features and prognosis of gastric adenocarcinoma have been clearly described. National Comprehensive Cancer Network (NCCN) guidelines present a detailed management strategy for advanced gastric adenocarcinoma. Previous studies have reported the 3-year survival rate to be 10% to 20% and the median overall survival (OS) was 11 months in patients with recurrent or metastatic gastric adenocarcinoma.^[5]^ However, primary gastric squamous cell carcinoma (SCC) is a rare type of GC, with a worldwide incidence of 0.04% to 0.4% of all GCs.^[6,7]^ The majority of studies investigating gastric SCC are limited to case reports. Therefore, there is a poor understanding of the clinical pathological features of SCC of advanced gastric SCC. The present study retrospectively reviewed and examined the clinical pathological features and prognosis of 14 patients with recurrent or metastatic gastric SCC between January 2003 and January 2016.

## 2 Methods

### 2.1 Patients

The present study retrospectively examined 14 patients with advanced SCC of the stomach, as confirmed by biopsy, including 7 patients from our hospital and 7 patient cases selected from the PubMed database. The inclusion criteria of the present study were as follows: histological diagnosis of gastric SCC; the tumor must not be located in the cardia area or extend into the esophagus; presence of local relapse or distant metastases of gastric SCC in computed tomography (CT) images; and no evidence of secondary SCC in the body. Clinical pathological data and follow-up data were obtained from the medical record or case report of each patient. These included age, gender, symptoms, laboratory, radiographic, operative and pathology reports, date of relapse/metastasis, chemotherapy records, survival time, and the date of mortality. The study was approved by The Institute Review Boards of Shandong Cancer Hospital.

### 2.2 Statistical analysis

The outcome measures used were OS (defined as the date from relapse/metastasis to mortality or cut-off date, January 30, 2016). Survival outcomes were estimated using the Kaplan–Meier method. All statistical analyses were conducted using Statistic Package for Social Science (SPSS) software, version 17.0 (SPSS, Inc., Chicago, IL).

## 3 Results

### 3.1 Clinical features

Fourteen patients (10 males and 4 females) with advanced gastric SCC were included in the present study. In total, 6 cases exhibited recurrent or metastatic lesions following radical surgical excision and 8 exhibited distant metastases at the time of diagnosis with gastric SCC. The median age of the 14 patients was 61 years old (range, 28–76). The clinical symptoms reported included upper abdominal pain (7 cases), weight loss (5 cases), dysphagia (2 cases), appetite loss (1 case), melena (3 cases), and hypotension (2 cases). Tumors were located in the lesser curvature of the stomach (8 cases), the greater curvature of the stomach (2 cases), the fundus of the stomach (1 case), posterior gastric wall (1 case), diffuse infiltrative carcinoma of the stomach (1 case), and the remnant stomach (1 case). A total of 2 patients exhibited a histologically well-differentiated SCC, 3 exhibited a moderately differentiated SCC, 6 exhibited a poorly differentiated SCC and the remaining 3 patients were unknown. The CT images indicated distant metastases of gastric SCC, with sites including the liver (9 cases), ovary (1 case), spleen (1 case), peritoneum (1 case), retroperitoneal lymph node (3 cases), and clavicle lymph node (1 case), with 3 cases exhibiting multiple organ metastases.

### 3.2 Treatment

Palliative chemotherapy was administered in 14 patients diagnosed with recurrent or metastatic gastric SCC (Table 1). The regimens of chemotherapy included docetaxel + oxaliplatin or cisplatin + fluorouracil (DOF) for 3 patients, fluorouracil, oxaliplatin + calcium folinate (FOLFOX) for 2, capecitabine + oxaliplatin (XELOX) for 2, docetaxel + capecitabine for 1, cisplatin + fluorouracil/campitabine/S-1 for 2, docetaxel + cisplatin/oxaliplatin (TP) for 3, gemcitabine + fluorouracil (GF) for 1 and pirarubicin + fluorouracil for 1 patient. The grade III–IV regimen-associated toxicity recorded during chemotherapy consisted of granulocytopenia in 2 cases, anemia in 2 cases, and vomiting in 3 cases. No grade III–IV toxicity information was recorded in the remaining cases.

**Table 1:**
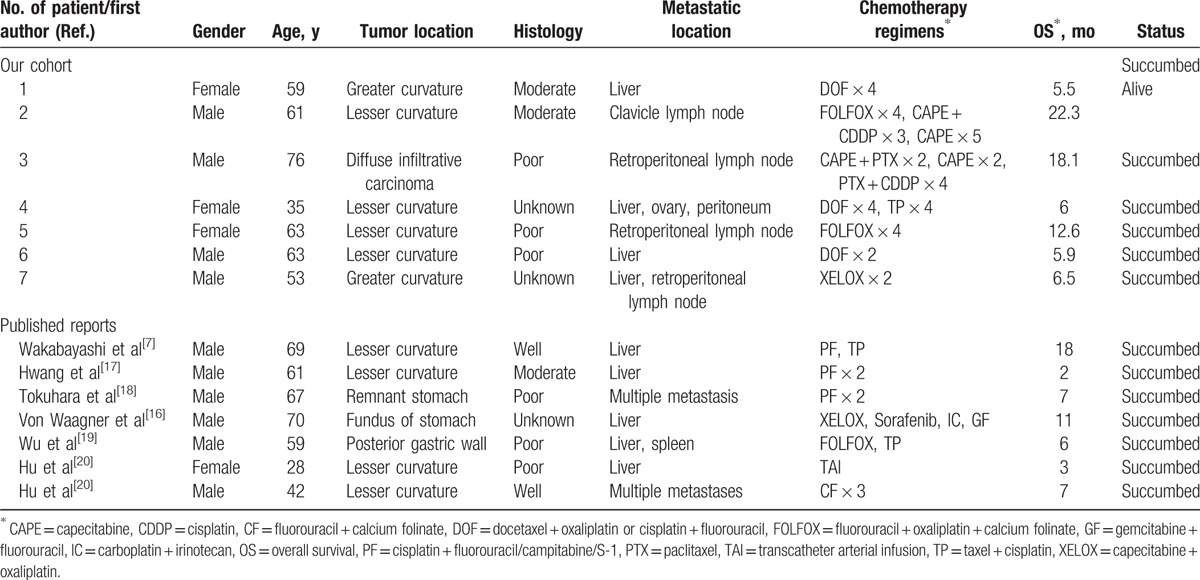
Characteristics of patients with recurrent or metastatic squamous cell carcinoma of the stomach.

### 3.3 Overall survival

**Figure 1:**
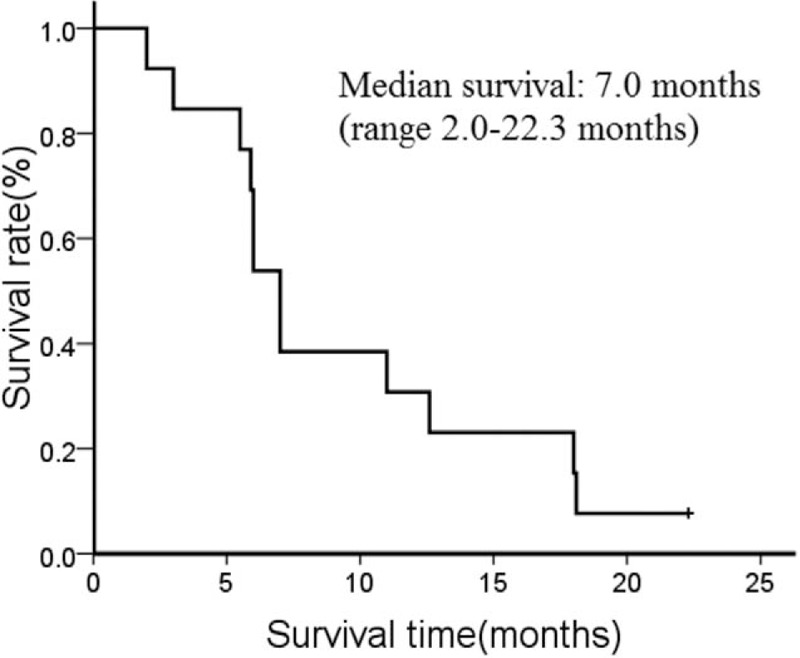
Survival of patients with advanced gastric squamous cell carcinoma.

Of 14 patients with advanced gastric SCC, 13 patients died of distant metastases and 1 case of hemorrhage of the digestive tract. In total, 1 patient survived to the follow-up cut-off date. The results of the meta-analysis for the 14 patients with advanced SCC of the stomach indicated that the median OS was 7.0 months (range, 2.0–22.3 months) (Fig. 1).

## 4 Discussion

Primary SCC of the stomach is a rare malignant gastric tumor. Approximately 100 cases have been previously reported, with the majority describing 1 single case.^[7,8]^ Numerous previous studies have provided conflicting evidence regarding the pathogenesis of gastric SCC, with the proposed mechanisms induced including: The formation of nests of ectopic squamous cells in the gastric mucosa; squamous metaplasia of the gastric mucosa before malignant transformation; squamous differentiation in a preexisting adenocarcinoma; and multipotential stem cells in the gastric mucosa.^[8–10]^

There are currently no clinical characteristics that enable patients with gastric SCC to be distinguished from patients with adenocarcinoma, with the diagnosis of gastric SCC currently confirmed by histopathological examination.^[11]^ To differentiate primary gastric SCC from the more common variants, 3 diagnostic criteria of the tumor must be met: not located in the cardia; not located in the esophagus; and no evidence of primary SCC elsewhere in the body.^[12]^ In the present study, the selected cases exhibited tumors that fulfilled these criteria, with a final diagnosis of primary gastric SCC. A previous study indicated that gastric SCC may involve any portion of the stomach.^[11]^ In the present study, 57% (8/14) of the tumors were located along the lesser curvature of the stomach, a location that is frequently associated with adenocarcinoma of the stomach.

Gastric SCC is rare and there is currently no consensus on how to treat this disease. Previous studies indicate that radical surgical excision can improve the prognosis of gastric SCC^[10]^ and is the only potential cure for localized disease.^[7,8]^ In the present study, 6 patients with gastric SCC who underwent radical surgical excision were observed to have a median progression-free survival of 10.0 months (calculated from the date of surgery to recurrence or metastasis) and a median OS of 15.9 months (data not shown), which may suggest that radical surgical excision may serve an important role in improving the survival of patients with gastric SCC.

Although the effects of chemotherapy on advanced gastric SCC have previously been described in case reports, only a few studies have demonstrated the efficacy of systemic chemotherapy against the recurrence or metastasis of primary SCC of the stomach.^[7,13]^ In the present study, due to the lack of standardized treatment strategies, a range of different chemotherapeutic regimens were administered to the 14 patients with advanced gastric SCC, including fluoropyrimidine-, platin-, and taxane-based combined chemotherapy.

The results indicated that the median survival time of patients with advanced gastric SCC was 7.0 months, which is shorter than the described 11.0 months of advanced adenocarcinoma of the stomach.^[5]^ This may suggest that patients with advanced gastric SCC have a poorer prognosis compared to patients with advanced adenocarcinoma of the stomach. However, this is difficult to conclude due to the limited nature of the present study. Furthermore, the poor outcome of the patients with gastric SCC may suggest that the standard chemotherapeutic regimens for gastric adenocarinoma are not suitable for patients with gastric SCC. Further study is required to investigate this.

Concurrent chemoradiotherapy has previously been demonstrated to exhibit improved efficacy in patients with nongastric SCC compared to patients with adenocarcinoma.^[14,15]^ Previous case studies have reported patients with gastric SCC exhibiting an OS of 27 months^[16]^ and 60 months^[10]^ following treatment with concurrent chemoradiotherapy.

In conclusion, the results of the current retrospective study demonstrated that the median survival time of patients with recurrent or metastatic gastric SCC was 7.0 months, which is shorter than the described 11.0 months of patients with recurrent or metastatic gastric adenocarcinoma. This suggests that patients with gastric SCC may have a poorer prognosis compared with patients with gastric adenocarcinoma. However, further clinical investigation is required to support this conclusion.

## Acknowledgment

Thanks to Medbanks (Beijing) Network Technology CO, Ltd for patient's follow-up.
